# Improving the Yields and Reaction Rate in the Ethanolysis of Soybean Oil by Using Mixtures of Lipase CLEAs

**DOI:** 10.3390/molecules24234392

**Published:** 2019-12-01

**Authors:** Margarita Díaz Ramos, Letícia Passos Miranda, Roberto Fernandez-Lafuente, William Kopp, Paulo Waldir Tardioli

**Affiliations:** 1 Postgraduate Program in Chemical Engineering, Department of Chemical Engineering, Federal University of São Carlos, Rodovia Washington Luís, km 235, 13565-905 São Carlos, SP, Brazil; margaritadiazramos@gmail.com (M.D.R.); lettypassos@gmail.com (L.P.M.); 2 Departmento de Biocatálisis, ICP-CSIC, Campus UAM-CSIC, 28049 Madrid, Spain; 3 Kopp Technologies (KTech), Rua Alfredo Lopes, 1717, Jardim Macarengo, 13560-190 São Carlos, SP, Brazil; kopp.tech.sp@gmail.com

**Keywords:** porcine pancreas lipase, *Thermomyces lanuginosus* lipase, mixture of CLEAs, soybean oil ethanolysis, vortex flow reactor, biodiesel

## Abstract

Due to the heterogeneity of oils, the use of mixtures of lipases with different activity for a large number of glycerol-linked carboxylic acids that compose the substrate has been proposed as a better alternative than the use of one specific lipase preparation in the enzymatic synthesis of biodiesel. In this work, mixtures of lipases from different sources were evaluated in their soluble form in the ethanolysis of soybean oil. A mixture of lipases (50% of each lipase, in activity basis) from porcine pancreas (PPL) and *Thermomyces lanuginosus* lipase (TLL) gave the highest fatty acid ethyl ester (FAEE) yield (around 20 wt.%), while the individual lipases gave FAEE yields 100 and 5 times lower, respectively. These lipases were immobilized individually by the cross-linked enzyme aggregates (CLEAs) technique, yielding biocatalysts with 89 and 119% of expressed activity, respectively. A mixture of these CLEAs (also 50% of each lipase, in activity basis) gave 90.4 wt.% FAEE yield, while using separately CLEAs of PPL and TLL, the FAEE yields were 84.7 and 75.6 wt.%, respectively, under the same reaction conditions. The mixture of CLEAs could be reused (five cycles of 6 h) in the ethanolysis of soybean oil in a vortex flow-type reactor yielding an FAEE yield higher than 80% of that of the first batch.

## 1. Introduction

Biodiesel is a promising substitute for petroleum-derived fuels [1,2,3,4,5,6]. Its current production is manly carried out via alkaline transesterification of fatty acid-free oils and alcohols [7]. An alternative to the alkaline catalysts is the use of immobilized lipases [3,4,8,9,10,11], which allows the use of oils containing a high percentage of free fatty acids as raw material, because these enzymes can catalyze both transesterification and esterification reactions in media containing low water activity [8,12,13]. Due to the heterogeneity of oils and fats, the use of a mixture of two or more lipases with different specificities has proved to be advantageous in the production of biodiesel. Several mixtures of lipases (mainly 1,3-especific and non-specific) have been reported as biocatalysts in biodiesel production, showing higher yields than those achieved using individual lipases [14,15,16,17,18,19,20,21], such as Lipozyme TL-IM and Novozym 435 in the methanolysis of rapeseed oil [22] and lard [23], lipases from *Rhizopus oryzae* and *Candida rugosa* immobilized on silica gel to catalyze the methanolysis of soybean [24,25,26] and canola oils [27], and 1,3-specific lipases from *Thermomyces lanuginosus* (immobilized in Lewatit VP OC 1600) and *Rhizomucor miehei* (commercial immobilized form, Lipozyme RM-IM) to catalyze the ethanolysis of soybean oil [28]. Even recently, it has been shown that a mixture of the same lipase immobilized following different protocols (this may alter the lipase specificity [29]) improves the final biodiesel yields [30].

Since the immobilization protocol plays a key role in the final biodiesel yields, as well as the final cost of the biocatalyst (the cost of the support itself and the cost of the technology involved in the immobilization process) [8,31], a carrier-free technique has attracted increasing attention [32,33,34,35]. This technique, called CLEAs (cross-linked enzyme aggregates), consists of the precipitation of the protein induced by the addition of a precipitant like salts, organic solvents, or polymers, followed by chemical crosslinking with bifunctional or poly-functional agents [32,36]. It has been considered a promising method for enzyme immobilization due to advantages such as high volumetric activity in the immobilized form, some enzyme stabilization, low production costs due to the absence of a pre-existing carrier, as well as the possibility of using a semi-purified enzyme and co-immobilizing different enzymes [31,37,38]. CLEAs of several enzymes, including many lipases, have been reported [33,37,39,40,41,42,43]. Some CLEAs of lipases have been reported as biocatalysts in the biodiesel production, as shown in Table 1.

However, as seen in Table 1, to the best of our knowledge, there are no reports on co-immobilization of lipases or mixtures of lipases immobilized by the CLEA technique used in organic synthesis.

In this context, this work aimed to evaluate the enzymatic synthesis of biodiesel catalyzed by a mixture of lipases individually immobilized by the CLEA technique. First, several free lipases were mixed at different activity ratios and evaluated in the transesterification of soybean oil using ethanol as acyl acceptor. Ethanol was chosen because its inactivating effect on the lipases is lower than methanol, avoiding the necessity of the addition of the acyl acceptor in successive steps in the reaction medium. Besides, in terms of renewability, the ethylic biodiesel (mixture of fatty acid ethyl esters; FAEE) is more sustainable as ethanol is produced biologically. Having chosen the lipases and the mixing ratio, they were individually immobilized by the CLEA technique. CLEAs of porcine pancreas (PPL) and *Thermomyces lanuginosus* lipase (TLL) were prepared as previously reported by Ramos et al. [42] and López-Serrano et al. [37]. Finally, the mixture of the CLEAs of lipases was used in the solvent-free ethanolysis of soybean oil. At the time reaction of maximum FAEE yield, the reuse of the mixture of CLEAs was also evaluated in a vortex flow reactor (VFR) operated like a stirred batch reactor. This kind of reactor is a good option when the catalysts are fragile but need to be homogenously suspended and mixed in the reaction medium, due to their gentle but efficient stirring system [53,54,55,56]. The reaction medium is confined in the gap between two concentric cylinders, the inner rotating and the outer generally stationary [54,57]. The agitation promoted by the inner cylinder is less aggressive than that one obtained with conventional stirrers [55,56]. 

## 2. Results and Discussion

### 2.1. Selection of Lipases

Table 2 shows the FAEE concentration achieved when using the individual lipases (Assays 1, 5, 9, and 13) and mixed non-immobilized lipases in the ethanolysis of soybean oil. Using individual lipases, the highest yield was obtained using *Pseudomonas fluorescens* lipase (PFL; less than 7.5 wt.%), while the lowest yield was obtained using PPL (less than 0.3 wt.%). Curiously, using different combinations of PFL with other lipases, which individually gave worse yields, improved the yields. Only by mixing PFL with *Candida Antarctica* Lipase B (CALB) the yields were clearly worse using the mixture of lipases than the individual ones. Another exception was the use of 25% PFL and 75% PPL. The improvement of the yields using mixtures of lipases was not universal but it was quite general, CALB being a general exception, i.e., very poor yields when used alone (1.90 wt.%) and mixed with TLL (1.77 wt.%, assay 12), PPL (1.22 and 1.64 wt.%, assays 17 and 18, respectively), and PFL (1.91 wt.%, assay 22), even when, individually, TLL and PFL gave better yields (4.32 and 7.42 wt.%, assays 1 and 9, respectively) than CALB. This shows the complexity of the process and the many phenomena that can be interacting simultaneously. The highest FAEE concentration (ca. 22 wt.%) was achieved using a mixture of TLL and PPL (50% of each lipase, in activity basis), while these lipases acting individually yielded very low FAEE yields (4.32 and 0.22 wt.%, respectively). The better reaction yield obtained with the mixture of lipases could be due to a synergic action of them over the complex mix of triglycerides that composes the soybean oil [14,15,19,26,28]. In order to confirm this synergic effect, a set of reactions were carried out using mixtures of lipases fully active or inactive.

**Figure 1:**
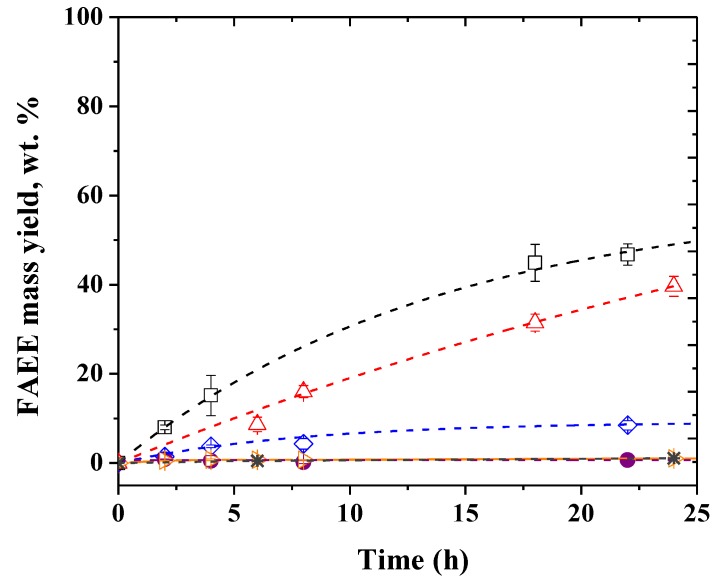
Time course of ethanolysis of soybean oil catalyzed by (□) mixture of PPL and TLL (total enzyme load of 500 U/g of oil); (∆) mixture of free TLL (fully active, 250 U/g oil) and PPL (fully inactive); (◊) free TLL (500 U/g oil); (●) free PPL (500 U/g oil); (▷) mixture of free PPL (250 U/g oil) and TLL (fully inactive); (*) mixture of fully inactive PPL and TLL. Reaction conditions: Soybean oil/ethanol molar ratio of 1:5 (10 g oil/2.63 g ethanol), 30 °C, and 300 rpm stirring (shaken flasks).

Figure 1 shows that after a 24 h reaction, the individual lipases TLL and PPL yielded an FAEE yield of only 9.0 and 1.1 wt.%, respectively, while the mixture of these lipases yielded a FAEE mass yield of 52.3 wt.%. Curiously, when a mixture of active TLL (250 U/g oil) and inactive PPL was used, an FAEE mass yield of 39.7 wt.% was reached, a yield 4.4-fold higher than that achieved when individual TLL (even using 500 U/g oil) was used and around 75% to that obtained using the mixture of active PPL and TLL. The PPL is supplied as a crude extract preparation containing a significant number of other hydrolases (esterases, amylases, and proteases) as contaminants; it also has some small components presented in pancreas and some protective agents for the lyophilization steps (e.g., starch) that may be added [58], some of which probably activated or even stabilized the TLL. However, the positive effect of the inactive PPL over the active TLL is lower than the increase of the yields using the mixture of active enzymes, which does not explain why the best yield was obtained using 1:1 of each enzyme preparation. Using PPL with inactivated TLL or just PPL, the yields were extremely low. Thus, we decided to continue the studies with PPL and TLL in the CLEA form, as this should eliminate the interferences generated by likely interactions between the contaminants of the PPL extract and TLL, because they will be immobilized together with PPL or washed away of the CLEA structure. Besides, even with PPL being an enzyme of animal origin, it has interest because it is cheaper compared to other commercial lipases [58].

### 2.2. Preparation of CLEAs of PPL and TLL

Changing sodium phosphate buffer [42] by hydrated ethanol in the step of the CLEA washing allowed obtaining CLEAs of PPL (named PPL–SOY–CLEA) with 100% immobilization yield and 89% expressed activity in the hydrolysis of tributyrin (the phosphate buffer washing left an immobilization yield of around 60% and an expressed activity around 40%) [42]. CLEAs of TLL prepared under the same conditions exhibited very low expressed activity in the hydrolysis of tributyrin (less than 10.4 and 16.3% using soy protein and bovine serum albumin as protein feeders; Table 3). The feeders were used to enhance the crosslinking step and also to reduce the mass activity of the biocatalysts, thus reducing diffusional limitations. This result shows that it was not viable to prepare combined CLEAs containing both PLL and TLL co-aggregated and crosslinked in the same supramolecular structure under optimal conditions. These kinds of problems are those that make co-immobilizing enzymes unadvisable unless it is strictly necessary, as many times the optimal protocol for one enzyme may not be the best for the other enzyme [59]. Moreover, it should be considered that the half-life of the co-immobilized catalysts will be dictated by the least stable enzyme [59]. This rule has some random exception, where the optimal protocol for CLEA formation is similar for both enzymes and their stabilities after immobilization are also similar [38]. Some strategies are being developed to overcome these problems [60,61,62], in some cases, specific for lipases [63]. 

Thus, CLEAs of TLL and PPL were prepared individually. For TLL, the protocol established by López-Serrano et al. [37] was used, using hydrated ethanol as precipitant. This CLEA, named TLL–SDS–CLEA, showed high immobilization yield (~100%) and high expressed activity, even a slight hyperactivation was observed (119%).

**Figure 2:**
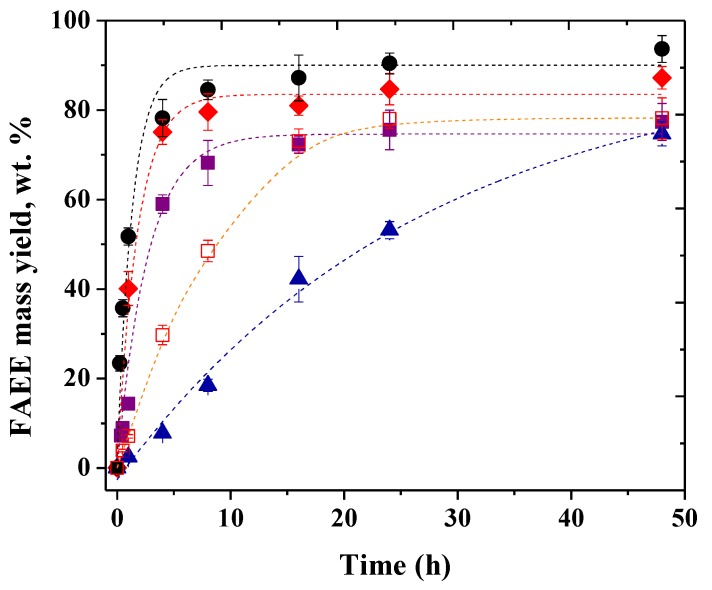
Time course of ethanolysis of soybean oil catalyzed by (▲) PPL–SOY–CLEA (3000 T/g oil); (■) TLL–SDS–CLEA (3000 U/g oil); (□)PPL–SOY–CLEA (6000 U/g oil); (♦) TLL–SDS–CLEA (6000 U/g oil); (●) mixture of PPL–SOY–CLEA and TLL–SDS–CLEA (3000 U/g oil for each CLEA). Reaction conditions: Soybean oil/ethanol molar ratio of 1:5, 30 °C, and 300 rpm stirring (shaken flasks). Reaction mixture content is given in Supplementary Materials (Table S1).

PPL–SOY–CLEA and TLL–SDS–CLEA were evaluated (separately and mixed) in the ethanolysis of soybean oil. Figure 2 shows that now PPL CLEA was able to produce a significant amount of biodiesel, while the non-immobilized enzyme was very inefficient in this reaction. The reaction rate doubled when the amount of enzyme doubled and a FAEE mass yield near 80 wt.% was achieved after 50 h. However, immobilized TLL remained more efficient than immobilized PPL. Rates almost doubled when doubling the amount of enzyme, and yields were slightly higher using more amount of enzyme after 50 h. The most significant result is that the reaction using the mixture of CLEAs of PPL and TLL exhibited an improvement in the FAEE production rate compared to that achieved using only TLL CLEA, mainly in the initial stage of the reaction (1 h) where the mixture of CLEAs gave a FAEE mass yield near 52 wt.%, while the TLL CLEA gave 40 wt.% yield. This means that at least part of the results obtained using the non-immobilized enzymes was due to a synergistic effect of the action of both lipases, and not only due to the positive effect of contaminants of the crude PPL extract in TLL. After a 48-h reaction, TLL CLEA (6000 T/g oil) gave a FAEE yield of 87 wt.%, while the mixture of both CLEAs gave a FAEE mass yield of 94 wt.%. That is, the simultaneous use of CLEAs of TLL and PPL permitted to increase the reaction rate and final yields, even though PPL CLEA give lower yields and reaction rates than TLL CLEA, as has been described in other papers that show the advantages of a mixture of lipases in this reaction [14,15,16,17,18,19,20,21,22,23,24,25,28]. In the case of a mix of PPL and TLL, the synergistic effect could be explained because PPL may exhibit higher activity versus some glycerol-linked carboxylic acids [64] that may behave as inhibitors for TLL, contributing to higher reaction rates. 

### 2.3. Influence of the Soybean Oil/Ethanol Molar Ratio in the Biodiesel Production

**Figure 3:**
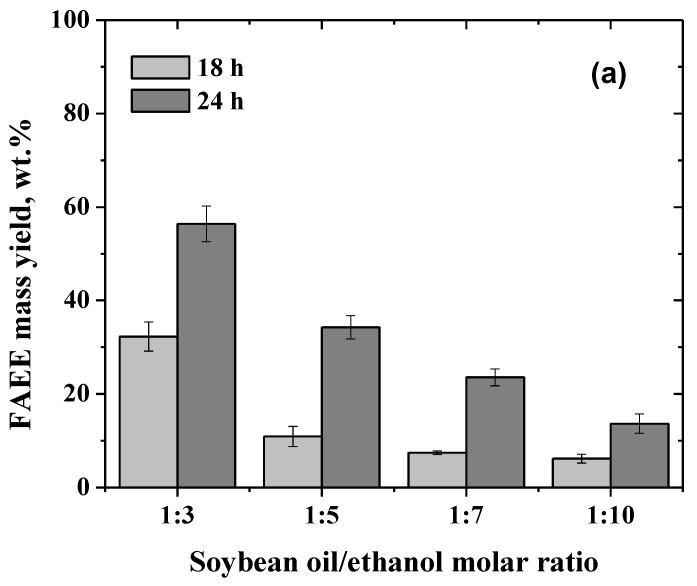

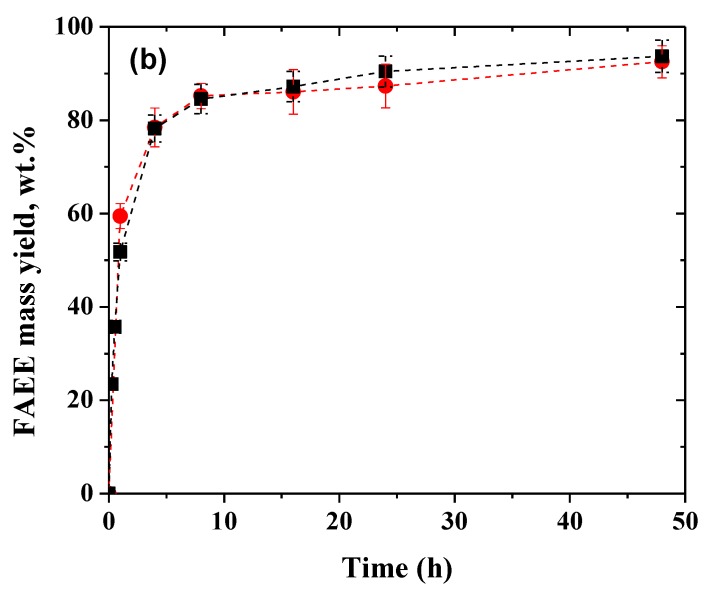
(**a**) Effect of soybean oil/ethanol molar ratio on FAEE mass yield obtained by the mixture of PPL–SOY–CLEA and TLL–SDS–CLEA (500 U/g oil) at 30 °C for 18 and 24 h. (**b**) Time course of ethanolysis of soybean oil catalyzed by the mixture of CLEAs (6000 U/g oil) at soybean oil/ethanol molar ratios of (■) 1:3 and (●) 1:5. In both assays, the stirring was kept at 300 rpm in shaken flasks. Reaction mixture content is given in Supplementary Materials (Tables S2 and S3).

Figure 3a shows that the highest FAEE mass yield (around 56 wt.%) was achieved under stoichiometric conditions. The excess of ethanol (from 3 to 10 moles/mol of soybean oil) led to a reduction in the reaction yield up to 4 times. This result may be explained by the ability of the ethanol to alter the hydration layer of enzymes, leading to an activity reduction or even to an enzyme inactivation [65,66,67]. 

**Figure 4:**
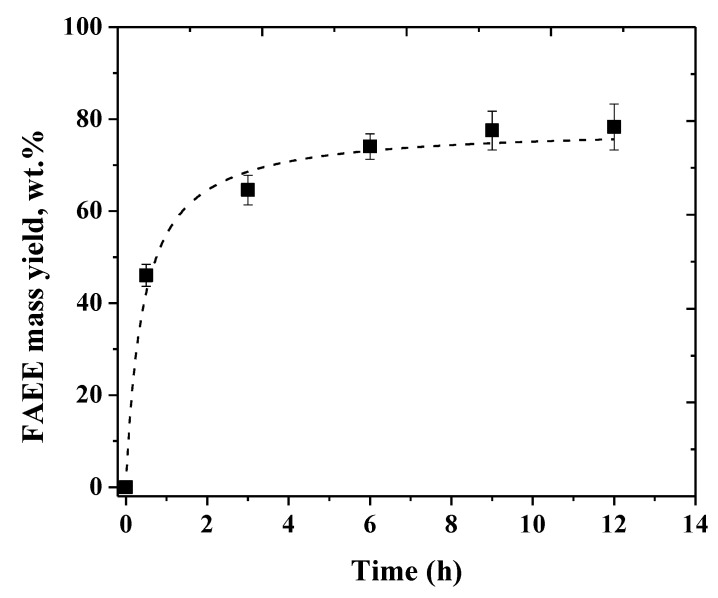
Time course of the ethanolysis of soybean oil catalyzed by the mixture of PPL–SOY–CLEA and TLL–SDS–CLEA in a vortex flow-type reactor. Reaction conditions: Enzyme load of 6000 U/g oil, soybean oil/ethanol molar ratio of 1:3, 30 °C, and a rotation of the inner cylinder of 900–2000 rpm. Reaction mixture content is given in Supplementary Materials (Table S4).

When soybean oil transesterification was monitored over time using two different oil/ethanol molar ratios (1:3 and 1:5) using 6000 U/g oil, no differences were observed in the course of FAEE production. Both reactions produced a FAEE mass yield of around 93 wt.% (Figure 4b). Thus, the stoichiometric oil/ethanol molar ratio was selected for further studies. 

### 2.4. Operational Stability of The Biocatalysts

The reaction course of the FAEE production by ethanolysis of soybean oil catalyzed by the mixture of CLEAs of TLL and PPL in a VFR operated as a stirred batch reactor is shown in Figure 4. In this reactor configuration, the FAEE yields were slightly lower than those achieved in the shaken flasks (Figure 3b). While in the latter, a FAEE yield of around 85 wt.% was achieved after 12 h reaction, in the former, around 80 wt.% was obtained. The main difference between the two reactor configurations evaluated was the stirring. In the shaken flasks, 300 rpm stirring was enough to perfectly suspend the CLEAs in the oil/ethanol mixture, but in the VFR, the stirring changed from 900 to 2000 rpm to suspend the CLEAs because the viscosity of the medium changed throughout the reaction. Besides that, when the reactor configuration is changed, differences in the heat and mass transfer profiles are expected, thus changing the reaction rates and, consequently, the product profiles. As the FAEE mass yield increased only around 4 wt.% for 6 h reaction (from around 74 to 78 wt.% when the reaction time increased from 6 to 12 h), the shortest time was chosen to evaluate the reusability of the biocatalyst. 

**Figure 5:**
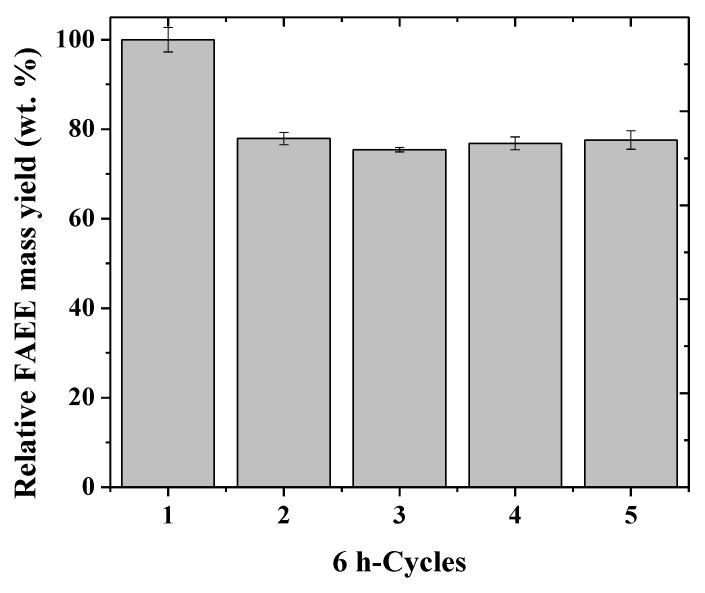
Reuse assay (6-h cycles) of the mixtures of PPL–SOY–CLEA and TLL–SDS–CLEA (6000 U/g oil) in the ethanolysis of soybean oil (molar ratio of 1:3) at 30 °C in a vortex flow-type batch reactor stirred initially at 900 rpm and gradually increased up to 2000 rpm. The FAEE mass yield (wt.%) at the first batch was taken as 100%. Reaction mixture content is given in Supplementary Materials (Table S4).

Figure 5 shows a decrease in the FAEE yield (wt.%) by around 22% from the first to the second batch, but the yield was well maintained in the other batches. The drop from the first to the second batch could be associated with loss of biocatalysts, mainly fine particles not recovered at the end of the first batch [68,69,70]. The loss of CLEA during washing and centrifuging has also been reported by Khanahmadi et al. [70]. They studied the reusability of CLEAs of a lipase extracted from cocoa pod husk (CPH) for biodiesel production and they observed that after seven 4-h cycles, the conversion percentage reduced to 58%. 

Regardless, the use of a mixture of CLEAs of TLL and PLL in the production of biodiesel in solvent-free medium showed to be an interesting strategy, which could be further optimized towards the operational stability of the biocatalyst. For example, treating the CLEA mixture with polyethyleneimine [71,72,73], aiming to prevent enzyme leaching and/or mass loss by shearing at very high stirring in batch reactors. 

## 3. Material and Methods 

Lipases from porcine pancreas type II (PPL; powder formulation with 237.6 ± 0.9 U/mg protein and 132.6 ± 0.5 mg protein/g), *Candida antarctica* (fraction B, CALB; liquid formulation with 1411.0 ± 10.1 U/mg protein and 9.3 ± 0.1 mg protein/mL), *Thermomyces lanuginosus* (TLL; liquid formulation with 2639.4 ± 32.1 U/mg protein and 23.1 ± 0.04 mg protein/mL), *Pseudomonas fluorescens* (PFL; powder formulation with 1096.0 ± 5.5 U/mg protein and 19.8 ± 0.1 mg protein/g), bovine serum albumin (BSA), *tert*-butyl alcohol, tributyrin, and Bradford reagent were purchased from Sigma-Aldrich (St. Louis, MO, USA). Glutaraldehyde solution (25% (*v*/*v*) in water) was purchased from Vetec Química Fina (Duque de Caixas, RJ, Brazil). Anhydrous ethanol (99.8%) was purchased from Synth (Diadema, SP, Brazil). Hydrated ethanol (96 GL) was acquired from a local gas station, and soy protein and soybean oil (Liza, Brazil) were acquired from a local market. All other chemicals and solvents were of analytical grade and were used as received. All assays were performed in triplicate and the values are expressed as mean ± standard error.

### 3.1. Biodiesel Production Using Different Free Lipases

Individual or mixed non-immobilized lipases were used to catalyze the ethanolysis of soybean oil at 30 °C for 8 h, according to the methodology described below. Four lipase preparations were mixed in six combinations (CALB/TLL, CALB/PFL, CALB/PPL, TLL/PFL, TLL/PPL, and PFL/PPL) and three activity percentage ratios (75/25, 50/50, and 25/75), and the fatty acid ethyl ester (FAEE) mass yields were quantified by gas chromatography. 

Having chosen the lipases to compose the mixture (in this case, TLL and PPL), one of them was fully inactivated before preparing the biocatalyst mixture, aiming to confirm the synergic action when both lipases were fully active. TLL was inactivated in a boiling water bath, and PPL was inactivated suspending the powder preparation in methanol and keeping for 1 h, and after drying at 60 °C for 24 h. The activity of these inactivated preparations was checked to be less than 1% of the native enzyme in the biodiesel production.

### 3.2. Preparation of CLEAs 

The CLEAs of PPL were prepared using soy protein as a feeder protein according to the methodology previously described by Ramos et al. [42], with minor modifications (changing phosphate buffer by ethanol in the step of the CLEA washing). A volume of 1 mL of a PPL solution (5.0 mg of protein/mL in 5.0 mM sodium phosphate at pH 7.0; the solution contained also soy protein to give PPL/soy protein mass ratios of 1:3) was added to 3.0 mL of hydrated ethanol in an ice bath. The resulting mixture was stirred at 150 rpm at 4 °C in an orbital shaker (Marconi, MA830, Piracicaba, SP, Brazil). After 30 min, the glutaraldehyde, 10 µmol of aldehyde groups/mg of total protein, were added and the suspension was left to react for 2.5 h. After centrifugation at 10,400× *g* for 10 min at 4 °C, the supernatant was discarded. Finally, in order to avoid lipase leaching from the CLEA structure during the washing step, the PPL CLEA was washed twice with 3 mL of the precipitating agent [37]—in this work, hydrated ethanol (96 GL). This CLEA will be hereinafter referred as PPL–SOY–CLEA.

On the other hand, the CLEA of TLL was prepared according to the methodology described by López-Serrano et al. [37], with minor modifications. One milliliter of a TLL solution (10.0 mg of protein/mL in 100 mM sodium phosphate buffer, pH 7.0, and 2.5% (*w*/*v*) of SDS) was added to 3.0 mL of hydrated ethanol in an ice bath. Then, 80 μL of 25% (*v*/*v*) glutaraldehyde was added. The resulting mixture was kept under 150 rpm stirring at 4 °C in an orbital shaker (Marconi, MA830, Piracicaba, SP, Brazil) for 16 h. One milliliter of hydrated ethanol was added to the final suspension to measure the hydrolytic activity before centrifugation. This procedure, except for the precipitating agent, was reported by López-Serrano et al. [37] as a way of measuring the CLEA activity before their recovery by centrifugation that can form large clusters, thus avoiding measuring the true activity of the CLEAs due to mass transfer problems. Then, the CLEA suspension was centrifuged at 10,400× *g* for 10 min at 4 °C, the supernatant was discarded, and the CLEA was washed twice with 5 mL of hydrated ethanol. The suspension was again centrifuged and the hydrolytic activity in the different supernatants was measured to calculate the immobilization yield. The CLEAs (hereinafter referred as TLL–SDS CLEA) were re-suspended in 5.0 mL of hydrated ethanol and their hydrolytic activity was again measured.

The immobilization yield was calculated as the ratio between the immobilized activity (i.e., total activity of the initial enzyme solution minus total activity of the washing supernatants) and the total activity offered to the immobilization (i.e., total activity of the initial enzyme solution). The expressed activity was calculated as the ratio between the observed activity of the CLEAs and the immobilized activity [42].

For further use in the ethanolysis reactions, the CLEAs of PPL and TLL were washed twice with anhydrous tert-butyl alcohol (in suspensions prepared at 20% (*w*/*v*) of CLEAs). The suspension was kept under gentle stirring at room temperature in an orbital shaker for 10 min. Then, the suspension was centrifuged at 10,414× *g* for 10 min, the solid phase was dehydrated in a refrigerator at 4 °C until constant mass, and the hydrolytic activities of the dried CLEAs were measured. A scheme of the preparation of CLEAs and the treatment of them to be used in the transesterification of soybean oil with ethanol is shown in Figure S1.

The hydrolytic activity of PPL–SOY–CLEA and TLL–SDS–CLEA were 33.40 ± 2.5 and 78.4 ± 2.6 U/mg of dry CLEA, respectively.

### 3.3. Ethanolysis of Soybean Oil

The reaction was started by addition of lipases. The activity ranged from an enzyme load of 500 U/g soybean oil (in the mixture of lipases, 250 U/g oil for each lipase) to a total enzyme load of 6000 U/g soybean oil (in the mixture of lipases, 3000 U/g oil for each lipase) and kept at 30 °C under 300 rpm stirring in an orbital shaker (Marconi, MA832, Piracicaba, SP, Brazil). Samples were periodically withdrawn, washed twice with boiling distilled water, centrifuged at 10,400× *g* for 10 min at 4 °C and dried at 60 °C prior to fatty acid ethyl ester (FAEE) quantification by gas chromatography. 

Several soybean oil/ethanol molar ratios (1:3, 1:5, 1:7, and 1:10) were evaluated. In this set of experiments, an enzyme load of 500 U/g soybean oil was used.

The ethanolysis of soybean oil carried out in sealed flasks and stirred in an orbital shaker was compared with that performed in the VFR [42] with the following specifications: Radius ratio (η = R_in_/R_out_) of 0.24, aspect ratio (Γ = L/d) of 6.72, and rotation of the inner cylinder (metallic rod coupled to an IKA overhead stirrer, IKA Werke GmbH & Co. KG, Breisgau, Germany) at 900 rpm stirring (necessary to obtain a homogeneous suspension of the CLEAs).

### 3.4. Operational Stability of The Mixture of CLEAs in The Synthesis of Biodiesel

The reuse of the mixture of CLEAs of PPL and TLL was evaluated in successive ethanolysis of soybean oil (oil/ethanol molar ratio of 1:3) carried out in the VFR described above. The reactor was operated at 30 °C for 6 h with a rotation of the inner cylinder of 900 rpm. A total enzymatic activity of 6000 U/g oil was offered. After each 6-h cycle, the reaction mixture was centrifuged to recover the CLEAs, followed by washing once with tert-butyl alcohol (in suspension prepared at 20% (*w*/*v*) of CLEAs) prior to being used in the next cycle.

### 3.5. Protein Concentration Determination 

Protein concentration was determined by Bradford method [74]. Bovine serum albumin (BSA) was used as a standard protein to construct a calibration curve.

### 3.6. Standard Activity Assay 

An amount of enzyme (100 μL of a CLEA suspension containing 2.5 mg of dry CLEA/mL ethanol) was added into the reaction medium composed of 6 mL of 0.1 M sodium phosphate at pH 7.5, 1.5 mL of tributyrin, and 16.5 mL of distilled water. The reaction mixture was incubated for 5 min at 37 °C. The hydrolysis reaction was monitored titrimetically in a Titrino 907 titrator (Metrohm, Herisau, Switzerland) using a 20 mM KOH solution. The hydrolytic activity was calculated from the amount of KOH consumed to neutralize the butyric acid released during the reaction [75]. One tributyrin unit (U) was defined as the amount of enzyme that releases 1 µmol of butyric acid per minute under the assay conditions.

### 3.7. Gas Chromatography

FAEEs were quantified by gas chromatography in a 7890A Agilent chromatograph (Santa Clara, CA, USA) equipped with a flame ionization detector according to the methodology described by Ramos et al. [42]. The FAEE mass yield (wt.%) was calculated according to Equation (1):

(1) $$\mathrm{FAEE}\;\mathrm{mass}\;\mathrm{yield}\;=\frac{\left(\sum A\right)-A_{\mathrm{EI}}}{A_{\mathrm{EI}}}\times \frac{C_{\mathrm{EI}}\times V_{\mathrm{EI}}}{m}\times 100\%$$

where ΣA is the total peak area of ethyl esters of fatty acids (C14:0 to C22:0); A_EI_ is the peak area of the ethyl heptadecanoate (C17); C_EI_ is the concentration of ethyl heptadecanoate (10 mg/mL); V_EI_ is the volume of ethyl heptadecanoate solution (1 mL); and m is the mass of sample (50 mg). FAME mix C14–C22 (Supelco) was used as standard for identification of ester peaks.

## 4. Conclusions

A mixture of free lipases (PPL and TLL, 50/50 tributyrin activity ratio) showed a synergistic action in the rate of ethanolysis of soybean oil. Both lipases immobilized by the CLEA technique under their respective optimized protocols permitted to achieve high immobilization yields and recovered activities, but the protocols were quite different, avoiding the use of the enzymes co-immobilized in the same CLEA supramolecular structure. The use of PPL CLEA greatly improved the yields in the production of biodiesel compared with the use of the free enzyme. The mixture of CLEAs was capable of catalyzing efficiently the transesterification of soybean oil with ethanol, improving the reaction rate and the FAEE yields when comparing with TLL CLEAs, even though PPL CLEAs gave much worse performance when individually used than TLL CLEA. Although for other kind of oils a study needs to be performed to choose the better lipases and the mixture of lipases composition (as also the oil composition will be different), this work showed that lipase mixtures are more efficient in the transesterification reaction than individual lipases, and when these enzymes are used in the immobilized form (here as CLEAs), the biodiesel mass yields are much more expressive. 

## Figures and Tables

**Table 1:** Cross-linked enzyme aggregates (CLEAs) of lipases ^a^ used as biocatalysts in biodiesel production.

Lipase CLEA	Immobilization Conditions	Reaction Conditions	Yield (%)	Reference
CLEAs of *Pseudomonas cepacia* lipase	Precipitant: Acetone 5 mg BSA/50 mg lipase 12-fold increase in the activity over the free enzyme powder	Jatropha seed oil/ethanol (1:4, mol/mol) CLEA (containing 6.25 mg lipase) 6 h reaction at 40 °C	90	[44]
		Mahua oil/ethanol (1:4, mol/mol) CLEA (containing 50 mg enzyme) 2.5 h reaction at 40 °C	92	[45]
CLEA of *Penicillium expansum* lipase	Precipitant: Ammonium sulphate The protein content in the CLEAs: 21 wt.%	Microalgal oil in the IL [BMIm][PF6]/methanol (1:3, mol/mol) 100 mg of CLEA 48 h reaction at 50 °C	85.7	[46]
CLEA of *Thermomyces lanuginosus* lipase (TLL)	Precipitant: Acetone Lipase/BSA mass ratio of 1:12 8-fold increase in the activity over the non-cross-linked lipase	Rapeseed oil or fish oil/ethanol (1:4, mol/mol) 10 wt.% CLEAs (with BSA in a mass ratio of 1:4) 1.5 h reaction at 40 °C The biocatalyst remained stable within 6 cycles of reuse	94 (Rapeseed oil) 68 (Fish oil)	[47]
CLEA of lipase B from *Candida Antarctica* (CALB)	Lipase/BSA mass ratio of 1:16 24-fold increase of the activity over the non-cross-linked lipase	Rapeseed oil or fish oil/ethanol (1:4, mol/mol) 10 wt.% CLEAs (with BSA in a mass ratio of 1:12) 24 h reaction at 40 °C	~80 (Rapeseed oil/fish oil)	
CLEA of lipase B from *Candida Antarctica* (CALB)	Precipitant: Ammonium sulphate Insolubilized CALB was covalently cross-linked to magnetic nanoparticles	Olive oil/2-propanol (1:6, mol/mol) 1 wt.% magnetic CLEAs within oil 24 h reaction at 30 °C Reuse in 10 cycles of 24 h without apparent loss of activity.	80 (92% after 72 h)	[48]
Multi-CLEA of lipase and protease from *Ictalurus punctatus* catfish viscera	Precipitant: Ammonium sulphate 0.113 mM of BSA Protease recovery activity: 43.82% Lipase recovery activity: 99.91%	Vegetable oil/ethanol (1:4, mol/mol) Enzyme-to-oil molar ratio: 1:10 Multi-CLEAs retained more than 34% of the initial activity after 5 batches for both enzymes	51.7	[49]
CLEA of *Thermomyces lanuginosus* lipase (TLL)	Tween 80 concentration: 1.0 mM 10 mg of amino- functionalized magnetite nanoparticles	Jatropha oil/methanol in isopropyl ether (1:3, mol/mol) CLEA (10 mg enzyme) 48 h reaction at 40 °C Tween 80-activated TLL- magnetic-CLEAs retained their activity during 10 cycles of 48 h	88	[50]
CLEA of pancreas porcine lipase (PPL)	Precipitant: Ethanol PPL: Soy protein mass ratio of 1:3 (The global yield was ~5-fold higher compared with standard PPL CLEAs) Immobilization yield ~60% Expressed activity ~40%	Soybean oil/ethanol (1:5, mol/mol) CLEA (50 mg enzyme) 24 h reaction at 30 °C fatty acid ethyl ester (FAEE) yield was higher than 50 wt.% within ten 24-h cycles of reuse	60	[42]
CLEA of lipase B from *Candida Antarctica* (CALB)	Precipitant: Ammonium sulphate Insolubilized CALB was covalently cross-linked to magnetic nanoparticles	Chlorella vulgaris lipids/methanol (1:10, mol/mol) 3 h reaction at 30 °C Magnetic CLEAs could be reused for at least ten catalytic cycles retaining 90% of the initial biodiesel conversion	87	[51]
CLEA of Km12 lipase	CLEA of Km12 was coupled with amino-coated magnetite nanoparticles Immobilization efficiency: 75%	Waste cooking oils/methanol (1:3, mol/mol) 0.3 wt.% immobilized lipase 36 h reaction at 35 °C Magnetic CLEAs retained its total activity up to 6 cycles of enzyme re-using in the standard assay condition	71	[52]

^a^ In some cases, CLEAs were prepared using a lysine-rich protein feeder (bovine serum albumin (BSA) or soy protein) to aid in the crosslinking step.

**Table 2:** FAEE mass yield of the ethanolysis of soybean oil with individual and mixed soluble lipases. Reaction conditions: 30 °C, soybean oil/ethanol molar ratio of 1:5 (10 g oil/2.63 g ethanol), shaken flasks at 300 rpm, 8 h reaction, and total enzyme load of 500 U/g oil.

Assay	Enzymatic Activity of Each Lipase in the Mixture (%)				FAEE Mass Yield, wt.%
	TLL	PPL	PFL	CALB	
1	100				4.32 ± 1.19
2	75	25			15.16 ± 1.72
3	50	50			22.03 ± 0.05
4	25	75			8.80 ± 0.33
5		100			0.22 ± 0.03
6	75		25		10.31 ± 0.19
7	50		50		11.50 ± 4.65
8	25		75		9.75 ± 2.36
9			100		7.42 ± 0.68
10	75			25	5.63 ± 0.39
11	50			50	4.36 ± 1.01
12	25			75	1.77 ± 0.87
13				100	1.90 ± 1.35
14		75	25		4.22 ± 2.23
15		50	50		9.97 ± 1.06
16		25	75		7.24 ± 1.03
17		75		25	1.22 ± 0.29
18		50		50	1.64 ± 0.80
19		25		75	2.20 ± 1.40
20			75	25	3.80 ± 1.27
21			50	50	3.53 ± 1.18
22			25	75	1.91 ± 0.16

**Table 3:** Synthesis of TLL CLEAs and combined CLEAs of TLL and PPL in the same conditions established for CLEAs of PPL [42] and described in Section 3.2. Two protein feeders were evaluated, soy protein (SOY) and bovine serum albumin (BSA).

CLEA	Lipase/Co-feeder Mass Ratio	Immobilization Yield (%)	Expressed Activity (%)
**TLL**	1:0	0	0
**TLL–SOY**	1:3	75.0 ± 0.1	2.9 ± 0.1
**TLL–BSA**	1:3	78.0 ± 1.0	15.8 ± 0.4
**TLL/PPL–SOY**	1:1	63.0 ± 1.0	1.9 ± 0.1
**TLL/PPL–SOY**	1:3	65.0 ± 5.0	10.37 ± 0.03
**TLL/PPL–SOY**	1:7	64.0 ± 0.1	11.2 ± 0.6
**TLL/PPL–BSA**	1:1	68.0 ± 2.0	1.34 ± 0.08
**TLL/PPL–BSA**	1:3	69.0 ± 1.0	16.3 ± 4.4
**TLL/PPL–BSA**	1:7	88.0 ± 1.0	15.5 ± 0.4

## Supplementary Materials

The following are available online: Table S1. Reaction mixture content in the transesterification assays shown in Figure 2; Table S2. Reaction mixture content in the transesterification assays shown in Figure 3a; Table S3. Reaction mixture content in the transesterification assays shown in Figure 3b; Table S4. Reaction mixture content in the transesterification assays shown in Figure 4 (time profile in the VFR) and 5 (reuse assays in the VFR); Figure S1. Scheme of preparation of CLEAs of porcine pancreas lipase (PPL) and *Thermomyces lanuginosus* lipase (TLL) for transesterification of soybean oil with ethanol.
