# Supplementary material for: Improving the Yields and Reaction Rate in the Ethanolysis of Soybean Oil by Using Mixtures of Lipase CLEAs
Source: Molecules. 2019 Dec 1;24(23):4392. doi: 10.3390/molecules24234392 (PMC6930585; doi:10.3390/molecules24234392)
Supplement: Supplementary file 1 [file molecules-24-04392-s001.pdf]

Supplementary Materials

Table S1. Reaction mixture content in the transesterification assays shown in Figure 2.

	U/g oil	Mass of CLEA (mg)	Total activity (U)	Oil (g)	Ethanol (g)
Mixture of CLEAs:<br>TLL-SDS-CLEA<br>PPL-SOY-CLEA	6000	25.02<br>88.36	6000	1.0	0.2812
PPL-SOY-CLEA	6000	176.72	6000	1.0	0.2812
	3000	88.36	3000	1.0	0.2812
TLL-SOY-CLEA	6000	50.05	6000	1.0	0.2812
	3000	25.02	3000	1.0	0.2812

Hydrolytic activities in U/mg dry CLEA: 119.89 (TLL-SDS-CLEA) and 33.95 (PPL-SOY-CLEA). All assays were carried using an oil/ethanol molar ratio of 1:5.

Table S2. Reaction mixture content in the transesterification assays shown in Figure 3a.

	Mass of CLEA (mg)			Mass in g	
Oil/ethanol molar ratio	PPL-SOY-CLEA	TLL-SDS-CLEA	Total activity (U)	Oil	Ethanol
1:3	37.42	15.94	2,500	5.0	0.7915
1:5	37.42	15.94	2,500	5.0	1.3192
1:7	37.42	15.94	2,500	5.0	1.8469
1:10	37.42	15.94	2,500	5.0	2.6385

Hydrolytic activities in U/mg dry CLEA: 78.4 (TLL-SDS-CLEA) and 33.4 (PPL-SOY-CLEA). All assays were carried out using 500 U/g oil.

Table S3. Reaction mixture content in the transesterification assays shown in Figure 3b.

	Mass of CLEA (mg)			Mass in g	
Oil/ethanol molar ratio	PPL-SOY-CLEA	TLL-SDS-CLEA	Total activity (U)	Oil	Ethanol
1:3	89.82	38.27	6000	1.0	0.1583
1:5	89.82	38.27	6000	1.0	0.2638

Hydrolytic activities in U/mg dry CLEA: 78.4 (TLL-SDS-CLEA) and 33.4 (PPL-SOY-CLEA). All assays were carried out using 6000 U/g oil.

Table S4. Reaction mixture content in the transesterification assays shown in Figures 4 (time profile in the VFR) and 5 (reuse assays in the VFR).

Mass of PPL-SOY-CLEA (mg)	Massa of TLL-SDS-CLEA(mg)	Total activity (U)	Mass of soybean oil (g)	Mass of ethanol (g)
779.22	382.65	60,000	10.0	1.5831

Hydrolytic activities in U/mg dry CLEA: 78.4 (TLL-SDS-CLEA) and 38.5 (PPL-SOY-CLEA). All assays were carried out using 6000 U/g oil, an oil/ethanol molar ratio of 1:3 and for 6 h reaction.

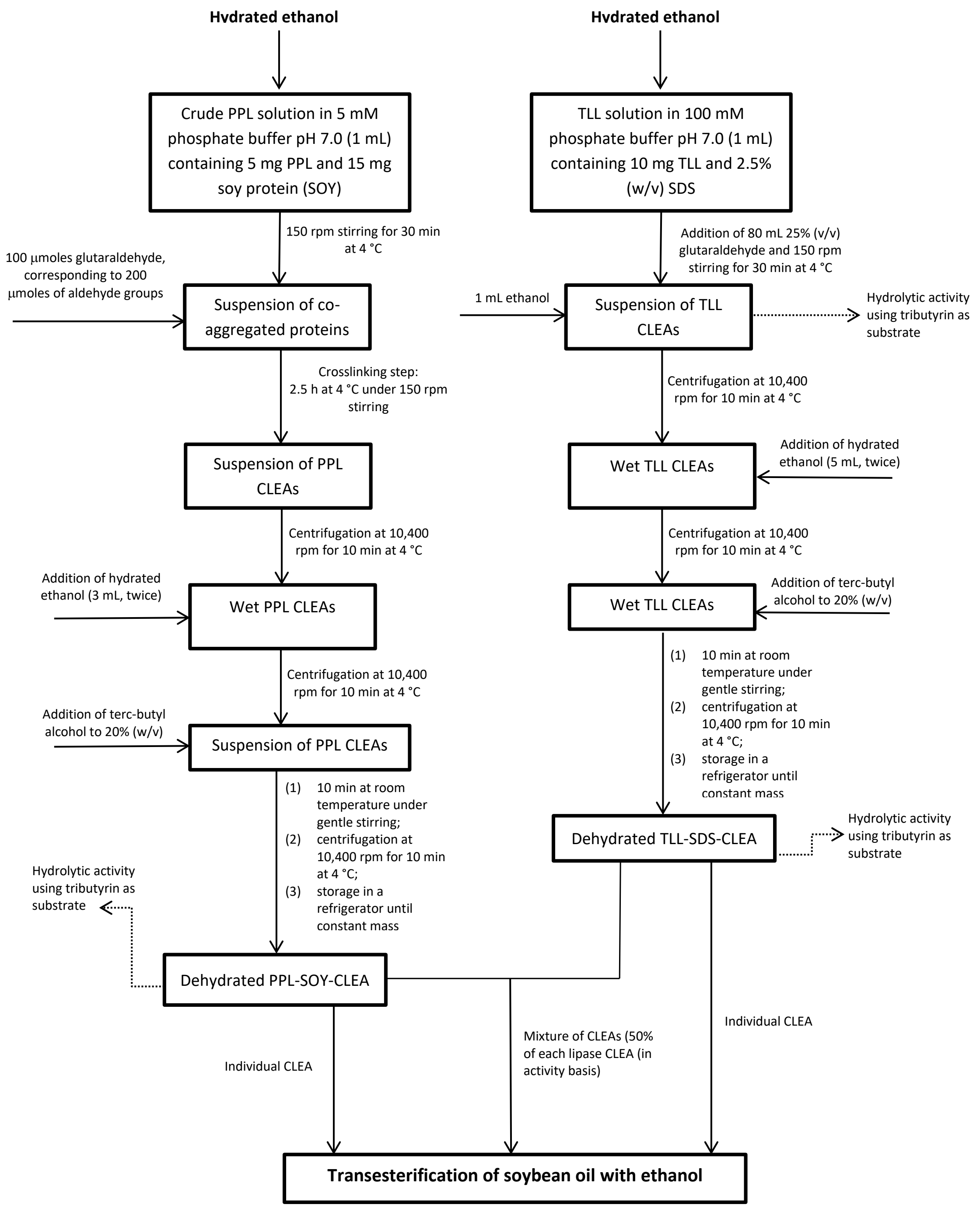

Figure S1. Scheme of preparation of CLEAs of porcine pancreas lipase (PPL) and *Thermomyces lanuginosus* lipase (TLL) for transesterification of soybean oil with ethanol.
